# *ShapeGuide*: Shape-Based 3D Interaction for Parameter Modification of Native CAD Data

**DOI:** 10.3389/frobt.2018.00118

**Published:** 2018-11-05

**Authors:** Yujiro Okuya, Nicolas Ladeveze, Cédric Fleury, Patrick Bourdot

**Affiliations:** ^1^VENISE Team, LIMSI, CNRS, Univ. Paris-Sud, Université Paris-Saclay, Orsay, France; ^2^LRI, Univ. Paris-Sud, CNRS, Inria, Université Paris-Saclay, Orsay, France

**Keywords:** virtual reality, 3D interaction, parametric CAD, part design, VR-CAD integration

## Abstract

While Virtual Reality (VR) technologies are commonly used in industrial companies, loading and modifying CAD parts of commercial CAD systems in virtual environments are still challenging. A few VR applications for Computer Aided Design (CAD) enable users to modify native CAD data in an immersive environment. Even if such VR-CAD applications use 3D interaction space, interaction with parameter values of CAD parts could be enhanced. This paper presents *ShapeGuide*, a technique allowing users to modify native CAD parts using a shape-based 3D interaction technique. With *ShapeGuide*, users can achieve modification of parameter values by directly pushing or pulling the surface of a CAD object. In addition, force feedback can be integrated into the technique to enhance the precision of the users' hand motions in the 3D space. In a controlled experiment, we compared *ShapeGuide* to a standard one-dimensional scroll technique to measure its added value for parametric CAD data modification on a simple industrial product example with an adjusted modification capability. We also evaluated the effect of force feedback assistance on both techniques. Results of this experiment demonstrate that *ShapeGuide* is significantly faster and more efficient than the scroll technique. Furthermore, they show that the force feedback assistance enhances the precision of both techniques, especially of *ShapeGuide*.

## 1. Introduction

The use of Virtual Reality (VR) technology for a product review is becoming common in industrial companies during the product development cycle: physical mockups are now replaced by digital mockups that let users assess the design of the product and conduct manufacturing simulations.

In the context of industrial design, products are necessarily modeled by parametric Computer-Aided Design (CAD) software to support the whole manufacturing process. While creating and modifying primitives and meshes using shape-based 3D interactions is possible in a virtual environment (Fiorentino et al., 2002; De Araújo et al., 2013), applying these interaction techniques on CAD data in such environment is challenging because of their complexities. Consequently, CAD engineers currently perform modifications on CAD object from workstations. To avoid this back-and-forth between VR systems and workstations, direct modifications of native CAD data in the virtual environment could improve the design process by reducing the number of iterations.

A previous study enabled to import and directly change parameter values of native CAD data in an immersive environment using one dimensional scrolling (Martin et al., 2017). However, this interaction technique was not consistent with 3D shape deformation because the modifications were achieved in parametric space. These parametric-based modifications often cause unpredictable shape deformation in 3D space which makes hard for non-CAD experts to reach the expected 3D shape.

Our goal is to enable users to modify native CAD data by a shape-based 3D interaction in an immersive environment. In addition, such interaction on the parametric model would enable non-CAD experts, as stylists or designers, to perform simple modifications on the CAD part by themselves.

In this paper, we propose *ShapeGuide*, a technique to modify native CAD data through a shape-based 3D interaction. *ShapeGuide* allows users to implicitly manipulate parametric constraints of CAD parts by grabbing and deforming its 3D shape. This technique prepares shape variations from an original design at run-time in order to guide the user hand gesture in 3D space. We compared the efficiency of *ShapeGuide* on parametric modifications of CAD data with a one-dimensional scroll technique in a CAVE-like system. In addition, we investigated the added value of force feedback assistance on these two interaction techniques.

This paper first review previous 3D interaction techniques in VR-CAD context. Sections 3 and 4 detail *ShapeGuide* and our VR-CAD framework. Then, sections 5 and 6 describe our controlled experiment. Finally, we discuss the results of the experiment and conclude with perspectives for future works in section 8.

## 2. Related work

Although 3D interaction techniques have been widely studied for immersive sketching and prototyping, it can hardly be applied to modifications of parametric CAD model. We first present the 3D interaction techniques of existing VR-CAD applications, then highlight the previous works for VR-CAD data integration, which attempted direct modifications of native CAD data during immersive product reviews.

### 2.1. 3D interaction for VR-CAD applications

Preliminary work on the VR-CAD system focused on carrying design activities from 2D to 3D space. Clark (1976), who demonstrated one of the first VR-CAD application using a prototype of HMD with a 3D wand, claimed: “*To expect a designer of 3-D surfaces to work with 2-D input and viewing devices unnecessarily removes a valuable degree of freedom.”* The studies in this field have mostly advanced since the 90s, many VR-CAD systems have been developed aimed at providing coherent dimension between visualization and interaction space. These applications can be classified broadly into two kinds according to targeted design activity: aesthetic design and solid modeling.

#### 2.1.1. Aesthetic design

Aesthetic design property is mostly considered at Conceptual design stage where stylists design the preliminary draft of the product. With the arrival of VR techniques, real pen and papers for sketching are getting replaced by digital tools.

Immersive drawing applications provide users one-to-one design capability of 3D objects. Israel et al. (2009) allowed the users to draw 3D lines in the air within a CAVE system using a 6DoF pen device. Fleisch et al. (2004) and Keefe et al. (2007) presented 3D drawing techniques inspired by tape-drawing, which is often used in automotive styling to easily create a full-size drawing or to highlight the design lines on clay models. More recently, a 3D drawing application with HMD, Tilt Brush, has been developed for artistic design (Skillman and Hackett, 2016).

These applications focus on free and preliminary drawing in 3D space, while some previous works presented geometric modeling applications. 3-Draw (Sachs et al., 1991) is the first application for free-form modeling using 3D interaction technique. The users can draw 3D wireframes by handling a stylus in 3D space. 3DM (Butterworth et al., 1992), 3DIVS (Kuester et al., 1999), and SpaceDesign (Fiorentino et al., 2002) presented surface modeling tools in 3D visualization spaces, by using a 6DoF mouse (Butterworth et al., 1992), a set of pinch gloves (Kuester et al., 1999) or the stylus (Fiorentino et al., 2002). 3DIVS and SpaceDesign enabled co-located interactions with digital mockups between the users' real hands and its visualization. Paljic et al. (2002) confirmed that manipulation of the digital mockups at a closer distance is significantly more efficient for the localization task in a 3D space. More recently, Mockup Builder (De Araújo et al., 2013) proposed the co-located bimanual finger interaction for rapid 3D prototyping.

The VR-CAD systems for aesthetic design put importance on direct 3D interaction with the digital mockup to reflect the users' creativity into the conceptual model. At a later product design stage, this model has to be built as a solid-model to consider its engineering feasibility for a manufacturing process design.

#### 2.1.2. Solid modeling

Many research works have attempted to change the interaction for solid modeling: from an alphanumeric input with mouse-based interaction to a direct shape-based interaction.

JDCAD (Liang and Green, 1994) is one of the first VR-CAD application in which users can create or edit primitives and perform Boolean operations by 3D interaction. JDCAD proposed *Region-based reshaping technique*: the users can manipulate specific parameters by dragging relevant control points mapped on the surfaces of the primitives. ARCADE (Stork and Maidhof, 1997; De Amicis et al., 2001) extended this approach and proposed a *Topological-context-based modification*: this technique considers not only the selected region on the surface but also the users' subsequent 3D gesture to determine the optimal object behavior according to users' hand stroke. However, it was difficult to meet all users' expectations for the object behavior from various users' input. Moreover, these works cannot address complex objects including geometric constraints, which makes more difficult to anticipate the object behavior.

To deal with such complex models, Gao et al. (2000) presented a 3D interaction technique to create and modify solid models containing different geometric constraints. Their VR-CAD system stores each primitive, parameters and operators within customized Constructive Solid Geometry (CSG), to recognize the related constraints from a selected element and enable consistent shape deformation with users' 3D hand motion. Ma et al. (2004) also tackled this issue with a hierarchical constraint-based data model. These approaches improved previous VR solid modelings to support constraint-based CAD models; however, the number of supported constraints and operators are limited, and they cannot directly load and save existing native CAD data since their approaches lay on custom data structures.

#### 2.1.3. Haptically aided design

Although 3D interaction techniques could enhance the intuitiveness of 3D design activities, many controversies surround precision of 3D hand gesture and fatigue of users' arms. Consequently, haptic feedback is often added to 3D interaction techniques to solve these issues and also to enhance the feeling of touch of virtual objects. Ye et al. (2006) compared the usefulness of each sensory feedback (vision, audio, and haptic feedback) on surface modeling task, and found that the haptic feedback marked the second highest place following the visual feedback.

Haptic feedback could be used in both aesthetic and solid modeling applications. For aesthetic design, Snibbe et al. (1998) and Gregory et al. (2000) provided the force feedback to drawing and painting activities in 3D space. Dachille IX et al. (1999) and Liu et al. (2004, 2005) presented surface deformation with pulling/pushing interaction using force feedback devices. For solid modeling, Picon et al. (2008) applied the force feedback to extrusion task to increase the 3D gesture accuracy.

### 2.2. VR-CAD data linkage

Immersive product reviews performed in many industrial companies do not involve any modifications of CAD data, and only experts perform all CAD data processing on workstations. In order to empower users to directly modify the CAD data and give them instant feedback in the immersive environment, some research works have challenged to integrate CAD systems and VR technology.

Schilling et al. (2006) presented a middleware framework in which the users can modify materials or textures on surfaces of the product during immersive reviews. This system was extended to support remote collaboration and to deal with heterogeneous VR platforms in Choi et al. (2010). VADE (Jayaram et al., 1999) and V-REALISM (Wang et al., 2010) can perform the assembly tasks based on native CAD data imported from Pro/Engineer® and Inventor® (Autodesk). These works succeeded to interact with CAD systems from immersive environments but did not address parametric modifications of CAD parts.

In previous VR-CAD systems, loading capability of existing CAD data designed with commercial CAD systems was often neglected. Only a few research works focused on VR-CAD data linkage for CAD part design in VR. One interesting approach is based on a labeling technique (Bourdot et al., 2010): a direct linkage between VR rendering of the Boundary Representation (B-Rep) of CAD objects with the nodes of the Constructive History Graph (CHG) of these objects. It aimed to allow an implicit edition of the CHG when users interact with B-Rep of the objects in the virtual environment. Martin et al. (2017) extended this model with an encapsulation technique to apply it on the CHG nodes and the B-Rep elements of most CAD systems used by industry. A proof of concept, named cRea-VR, has been implemented onto CATIA V5® (Dassault Systèmes), allowing the users to implicitly access parameter values of relevant CHG nodes from a surface selection in the virtual environment. This study enabled direct CAD part parameters modification during immersive product reviews using a simple one-dimensional scrolling interaction technique: increasing or decreasing parameter values.

### 2.3. Summary

Effective co-located 3D interaction techniques have been studied for aesthetic design, and some research works attempted to apply it onto solid modeling. Yet, these interaction techniques have not been used to live modifications of native CAD data in an immersive environment despite some recent advances in VR-CAD integration (Martin et al., 2017). As the data structure of CAD objects can be complex and depends on CAD systems, it is difficult to determine the object deformation from a given parameter change.

The *ShapeGuide* technique, described from next section, takes up this challenge.

## 3. *Shapeguide* methodology

While parameter oriented interaction techniques meet CAD user needs, such interaction does not fit to non-CAD experts. Coffey et al. (2013) developed an interface in which designers could refine the design of a medical device built with SolidWorks® by directly dragging the parameterized surface or simulation results (e.g., FEA, CFD) in 2D space. This study allowed designers to avoid interacting with parameter values but with the simulation results to modify CAD data. Similarly to their concept, shape-based 3D interaction techniques could provide non-CAD experts more straightforward interaction method and empower them to perform modifications of native CAD data during immersive product reviews.

In such review meetings, different industrial experts would collaborate to slightly edit constrains parameter values to refine the shape following an already chosen design intent based on the artistic and engineering criteria. In that case, complete edition of the original CAD model, such as geometric constrain creation or deletion should not be performed. Our focused scenario would be the design meeting where experts can edit parameters of the CAD model without CAD expertise to discuss the final design in a realistic immersive environment.

In this section, we describe a new 3D interaction technique, namely *ShapeGuide*, that computes shape variations to guide the users' hand motion during the CAD part deformation. With this approach, we can slightly anticipate the CAD object behaviors from each parameter selection to enable users to modify the part with a co-located pulling/pushing interaction on the shape. In the following, we detail the methodology of *ShapeGuide* and the computation of force feedback guidance.

### 3.1. Mesh computation

**Figure 1:**
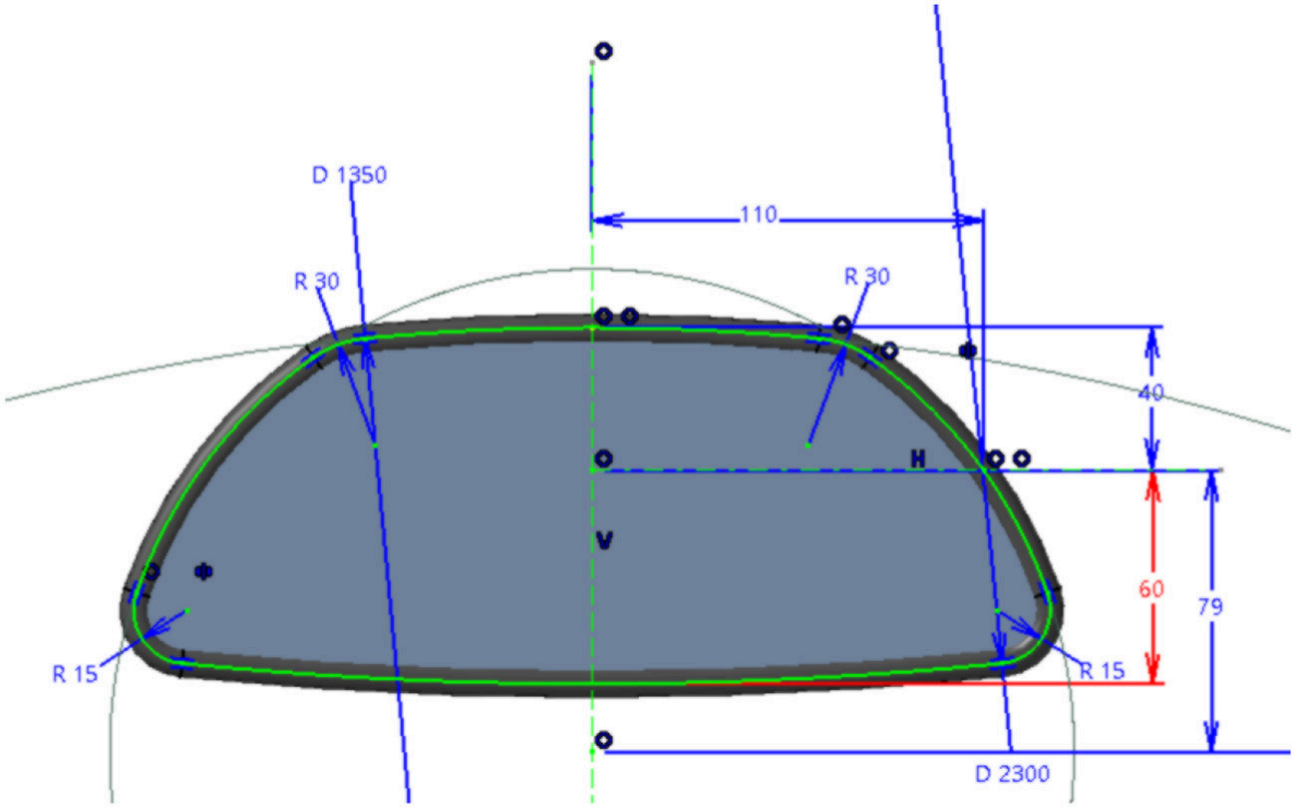
Sketch of the *Rear-view mirror*, an example of industrial CAD part designed using CATIA V5®. Green line is a guide curve of a sweep operation generating the 3D shape. Constraints and parameter values are highlighted in blue: for example, red constraint defines the distance to the bottom of the *Rear-view mirror* (60 mm).

The main difficulty of a 3D shape-based interaction with a constraint-based CAD object is the “unpredictability” of the shape deformation from a given parameter change. In parametric CAD systems, 3D models are usually defined by a set of operations (e.g., Extrusion, Sweep, Boolean operations) applied from primitives and 2D sketches, based on many parameters and geometrical constraints. Figure 1 is an example of real industrial CAD model designed by CAD engineers of an automotive company: the *Rear-view mirror* of a car. This *Rear-view mirror* is generated from a Sweep operation following a guide curve (Figure 1, green lines), which is defined by different parameters (radius, lengths) and geometric constraints (tangency and symmetry).

**Figure 2:**
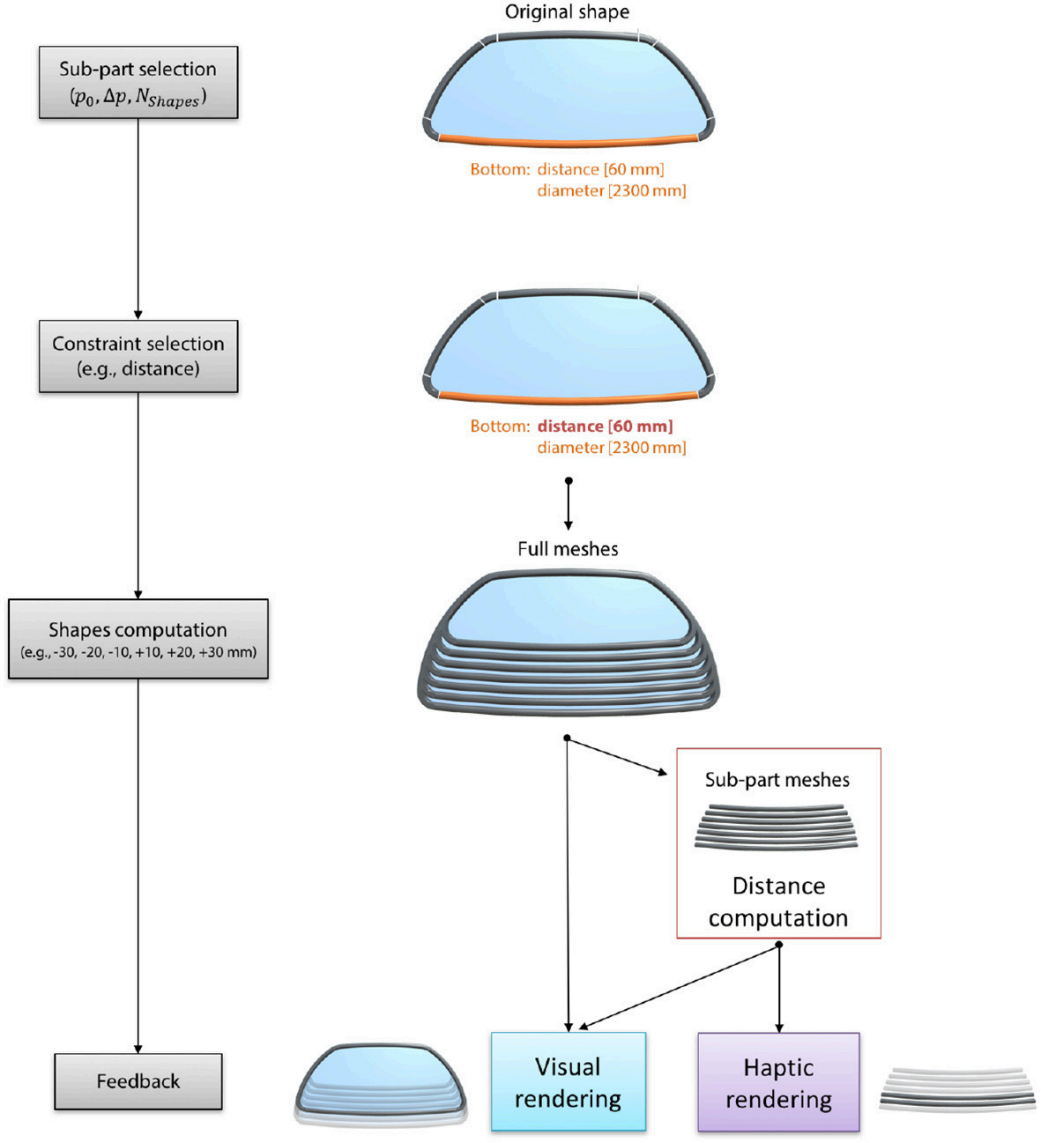
Computation of different shapes of *Rear-view mirror*: when users select the red distance constraint highlighted in Figure 1, full meshes are used to provide visual feedback to the users (only one mesh is visible at a time), and sub-part meshes are used for haptic rendering (the two closest meshes are used for force feedback computation).

To modify parameter values of such complex CAD objects with a shape-based interaction, we compute several possible shapes from a set of discrete parameter values. For example, when the user selects the bottom part of *Rear-view mirror* in a virtual environment, our VR-CAD system uses VR-CAD data linkage to list constraints related to the bottom part (i.e., distance: 60 mm and diameter: 2,300 mm). After the user selects the constraint, the system computes several shapes from variations of the corresponding parameter value (Figure 2). Due to tessellations of its B-Reps, *Rear-view mirror* is composed of nine sub-part meshes. While we render the complete *Rear-view mirror* (full meshes in Figure 2) for visual feedback, only sub-part meshes are used for physics computation to reduce computation cost. For this physic rendering, the distance between the user's hand and sub-part meshes are computed at each frame for both visual and haptic rendering. For the visual feedback, only the closest full mesh is rendered in the virtual environment to make the user feel that the CAD object is deformed through a pushing/pulling interaction regardless of its hidden internal geometric definition. Haptic feedback can be computed from distances between the user's hand and closest two meshes to assist the shape selection process as described in section 3.3.

Tessellation of B-Reps takes time according to CAD model size and complexity. In the *ShapeGuide* methodology, sub-part meshes generation impose a loading time after each selection to guarantee a real-time interaction while the user is modifying a parameter value. While complete update could be achieved on primitives or simple CAD object in real-time, updating a complex CAD model at each frame during parameter modification could delay system response that strongly impacts user's performances. Indeed, the real-time sensory feedback to users (60 Hz for visual feedback and 1 kHz for haptic feedback) is one of the critical criteria to maintain their immersion in a virtual world. Even if *ShapeGuide* requires some loading time, it ensures real-time system responsiveness during modifications for any CAD model regardless of its complexity. As an example, tessellations of the six sub-part meshes of the bottom part of *Rear-view mirror* presented in Figure 2 takes between 0.9 and 1.2 seconds.

The number of shapes *N*_*shapes*_ and parameter step size Δ*p* need to be specified at sub-part selection stage to define the parameter set surrounding current parameter *p*_0_ of the constraint. These values can be changed at each selection during the simulation to be able to set any parameter value with the required precision. After the sub-part selection, users can choose the constraint to modify if more than one constraints are linked to the selected part. In the current system, we did not implement a user interface to allow the user to tune these values, *N*_*shapes*_ and Δ*p* as well as the one to select the constraint within the list fetched by VR-CAD linkage. For the experiment purpose described in section 5, we did not let users manipulate these values during the experiment. Instead, we imposed static values for *N*_*shapes*_ and Δ*p* and specified a default modifiable constraint for each sub-part to control the loading time fitting our experimental hardware capabilities and time limitation for immersive experiments.

### 3.2. 3D shape-based interaction

**Figure 3:**
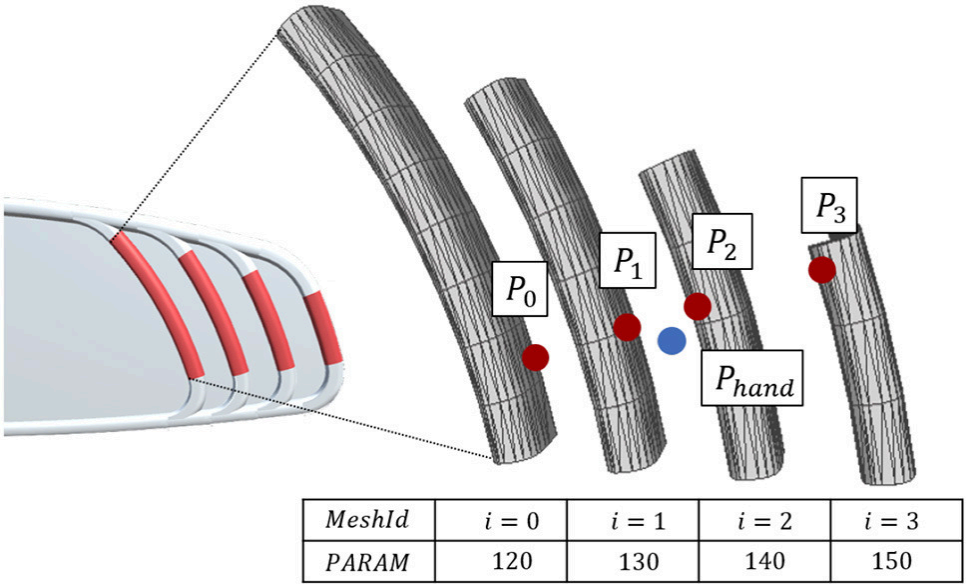
Example of computed sub-part meshes of the right side part for 3D interaction. *P*_*hand*_ is user's hand position. *P*_*i*_ is a nearest point on each surface from *P*_*hand*_.

Once the shapes are computed from a given parameter set, users can select one of them from their 3D hand position *P*_*hand*_ (Figure 3). Algorithm 1 performs this selection by computing each nearest point *P*_*i*_ and minimal distance *D*_*i*_ on each mesh within a set of generated sub-part meshes (*MeshSet*). *Nearest*3*DPointOnMesh*(*M, P*) in Algorithm 1 computes the nearest point on each triangle of a *M* mesh from a given *P*(*x, y, z*) position based on the computation algorithm described in (Ericson, 2004, p. 141-142). Then it returns the two closest points (*P*_*closestA*_ and *P*_*closestB*_) and nearest mesh Ids (*MeshId*_*A*_ and *MeshId*_*B*_). After this computation, the closest mesh Id (*MeshId*_*A*_) is sent to VR simulation to display the selected nearest shape in a virtual environment.

**Algorithm 1:** Two Closest Sub-parts Selection

**Input:** (*MeshSet*[], *MeshSetSize*, *P*_*hand*_) *Initialization*
1: *D*_*minA*_ ← Nearest3DPointOnMesh(*MeshSet*[0],*P*_*hand*_)
2: *D*_*minB*_ ← *D*_*minA*_ *LOOP Process*
3: **for** *i* = 1 to *MeshSetSize* **do**
4: (*P*_*i*_, *D*_*i*_) ← *Nearest*3*DPointOnMesh*(*MeshSet*[*i*],*P*_*hand*_)
5: **if** (*D*_*i*_ ≤ *D*_*minB*_) **then**
6: **if** (*D*_*i*_ ≤ *D*_*minA*_) **then**
7: (*MeshId*_*B*_, *P*_*closestB*_) ← (*MeshId*_*A*_, *P*_*closestA*_)
8: (*MeshId*_*A*_, *P*_*closestA*_) ← (*i*,*P*_*i*_)
9: **else**
10: (*MeshId*_*B*_, *P*_*closestB*_) ← (*i*,*P*_*i*_)
11: **end if**
12: **end if**
13: **end for**
**Output:** *MeshId*_*A*_, *MeshId*_*B*_, *P*_*closestA*_, *P*_*closestB*_

### 3.3. Force feedback assistance

An additional benefit of *ShapeGuide* is to be able to convey haptic feedback while modifying CAD part. The force feedback model is inspired by force feedback grid (Yamada et al., 2002), which stabilizes user's hand onto attractive points distributed on Cartesian axis. As a visual proxy comes close to the attractive point, the amount of the force becomes higher. Yamada et al. (2002) explain this force concept as “*This is analogous to the force by which a piece of iron would be attracted to a magnet.”*

**Figure 4:**
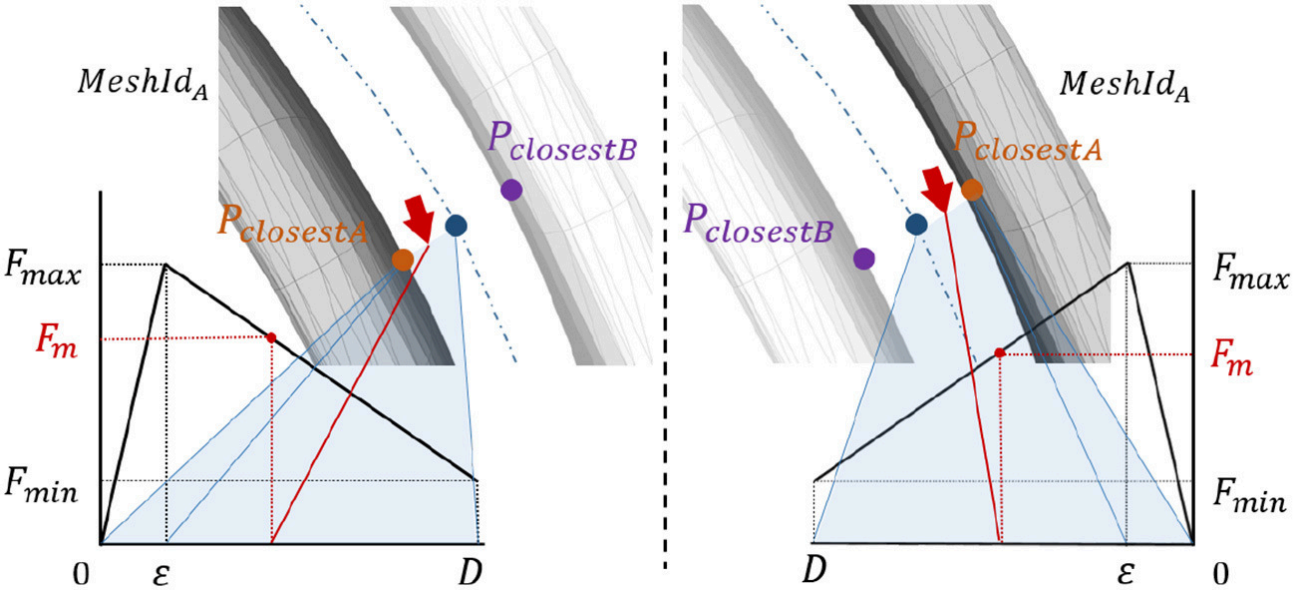
Amount of the attractive force feedback during modification of a shape. Red arrows describe user's hand position.

We extended this magnet metaphor from homogeneous Cartesian grids to arbitrary axis in 3D space. This force feedback attracts the user's hand onto the surface of the nearest sub-part mesh during the shape edition to hold the user's hand steady and to guide the hand toward neighbor meshes. The attractive point on the surface (*P*_*closestA*_) is acquired from the distance computation process (Algorithm 1). Then, the amount of magnetic force *F*_*m*_ (Figure 4) is computed as:

(1) $$F_{m}=\{\begin{array}{ll} F_{max}*\frac{d_{min}}{\epsilon } & \;d_{min}\in ]0,\epsilon ]\\ (F_{max}-F_{min})*\frac{D-d_{min}}{D-\epsilon }+F_{min} & \;d_{min}\in [\epsilon ,D[ \end{array}$$

The distance *D* is the half distance between the two closest shapes:

(2) $$D=||\vec{P_{closestB}P_{closestA}}||/2$$

*F*_*max*_ is the maximum force value, and *F*_*min*_ is the continuous force applied on the user's hand independently of his position. The force is modulated by the velocity of the user's hand with dumper model in order to avoid the vibrations nearby *p*_*closestA*_.

(3) $$F_{d}=-b*\frac{\text{d}}{\text{d}x}d_{min}\;(b>0)$$

*b* is a viscosity coefficient used for force feedback stabilization. Thus, the force feedback norm *F* is defined as:

(4) $$F=F_{m}+F_{d}$$

## 4. VR-CAD system architecture

**Figure 5:**
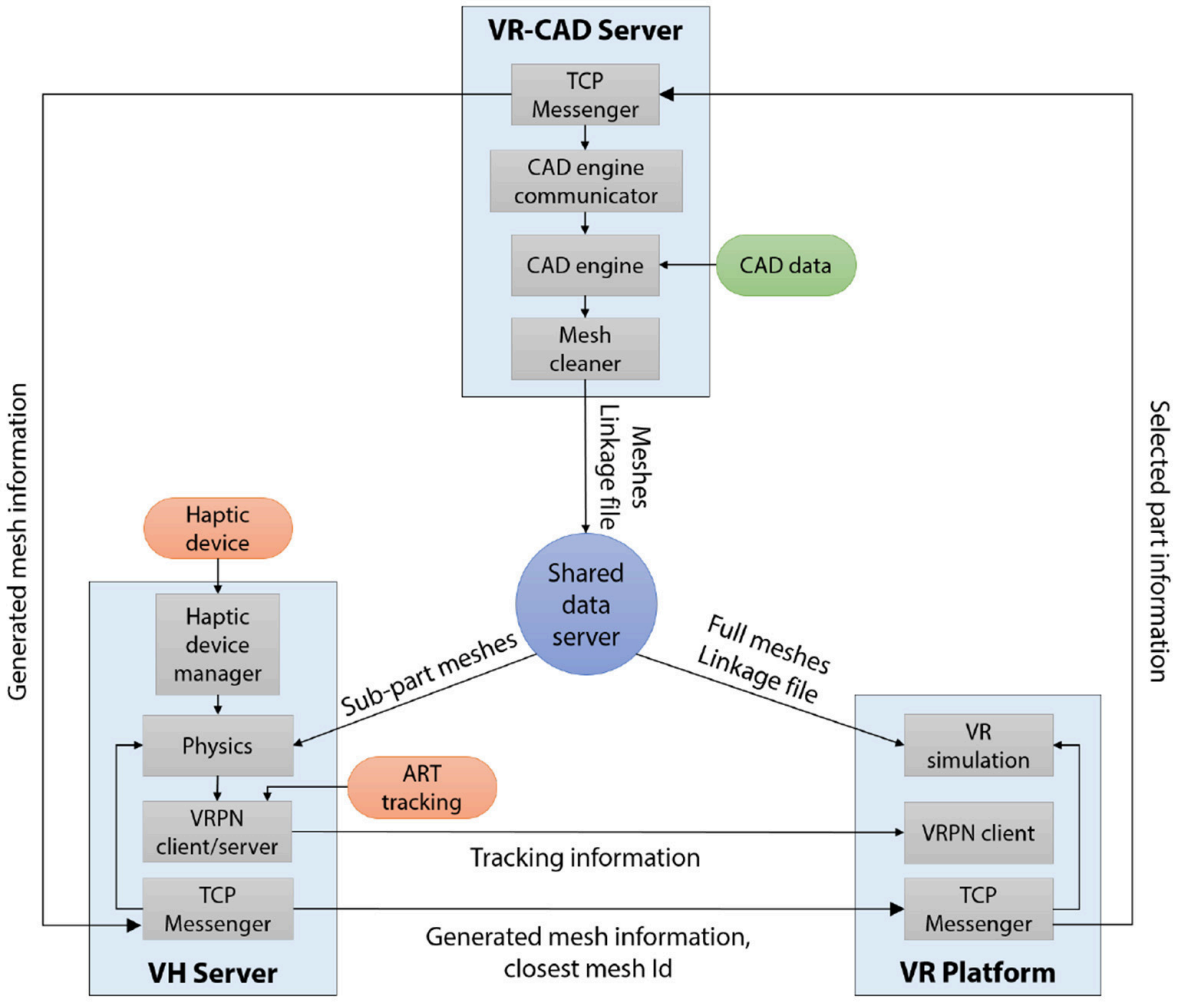
System architecture of our VR-CAD system. Each process is connected through TCP protocol: meshes and linkage information of CAD data are distributed using *Shared data server*. Selected part information contains: sub-part Id, constraint Id and a set of parameter offsets. Generated mesh information includes: number of generated meshes and parameter values.

The *ShapeGuide* technique lies on the VR-CAD system in which the users can directly modify native CAD data. Our VR-CAD system combines a core system architecture of previous work with a client/server architecture close to the Wang et al. approach (Wang et al., 2010). The system is composed of CAD engine communicator (*VR-CAD Server*), interaction manager (*VH Server*) and immersive visualization system (*VR Platform*). This distributed system (Figure 5) can run each process with different frame rates to manage physics computation during VR rendering, and also support heterogeneous visualization systems.

### 4.1. VR-CAD server

In order to deal with native CAD data in an immersive environment, the system needs to interact with a CAD engine in charge of loading original CAD source file and update any parameter modifications into the CAD data structure. To do so, we extended the cRea-VR approach proposed by Martin et al. (2017) to make it functional in the distributed architecture, namely *VR-CAD Server*. Initially, cRea-VR can parse and retain CHG data of native CAD data using CAA V5 (API for CATIA V5®) and tessellate its B-Rep elements in an immersive virtual environment.

When the system starts, *VR-CAD Server* loads a specified CAD data (.catpart) and generates related meshes and the linkage information (.xml), which describes the links between each mesh and CHG nodes of the CAD data. This file can be used as a reference to send modification request from *VR Platform* to *VR-CAD Server*. When users select a sub-part in the immersive environment, *VR-CAD Server* receives information of selected sub-part (sub-part Id and constraint Id) and new parameter set for the targeted constraint, then output the computed meshes. If a given parameter value is “invalid” because of unsolvable constraints issue, the related mesh is not generated. The output meshes tessellated by CAD systems may have deficits (e.g., non-manifold geometry), we therefore clean the meshes using VCG library^1^ and convert to Wavefront standard format (.obj) before deployment.

For example, when users select the distance of the bottom part of *Rear-view mirror, VR-CAD Server* asks CAD engine for parameter edition of the specified CHG node (distance = 60 mm on Figure 1, highlighted with red line) with new parameter values. Then, the CAD engine updates the whole CHG (e.g., relevant operators) and generate new meshes from the computed B-Reps.

### 4.2. Distributed system architecture

Our distributed architecture manages VR simulation by peer-to-peer connection among each process with centralized CAD data distribution. Each process in our VR-CAD system runs on a different computer, communicating through TCP protocol. *VR-CAD Server* deploys required information to interact with the CAD data (meshes and linkage file) to *VH Server* and *VR Platform* using a *Shared data server*.

*VH Server*, an acronym of a Visuo-Haptic Server, lies on two main parallel threads: physics and VRPN Client/Server. In the physics thread, the distance computation described in section 3.2 is managed between user's hand (i.e., haptic arm) position and the computed sub-part meshes loaded from *Shared data server* at each haptic frame. The physics thread also handles force feedback computation. VRPN Client/Server compounds visual proxy position with motion tracking data gathered from an external VRPN server, and broadcasts it to *VR platform* for visual rendering.

*VR Platform* manages an immersive visual rendering and handles events during the VR simulation. When the rendering system is composed of a set of clusters, only graphic master node (master of synchronization node) handles communication with *VH Server* before deploying the meshes to graphic slaves.

### 4.3. Data flow of *shapeguide*

When a user selects a sub-part of the original shape in a virtual environment, our system runs following processes until the user validates the modification. Step 3' and 4' are iterative processes while the user switches meshes computed based on the user's selection information.

1. **Part selection:** *VR Platform* finds constraint Id related with the selected sub-part using a linkage file. After the constraint selection, it transmits selection information (part Id and constraint Id) with a list of *N*_*shapes*_ and parameter step size Δ*p* from the current parameter value *p*_0_ to *VR-CAD Server*.
2. **Shape computation:** *VR-CAD Server* computes and generates several meshes of the selected sub-part based on the given parameter set. All generated meshes are saved to the *Shared data server*.
3. **Physics computation:** *VH Server* imports the sub-part meshes from the *Shared data server* to compute the nearest distance between the meshes from the user's hand position. The computed distance is used for haptic rendering, and the closest mesh Id is transmitted to *VR Platform* to switch the current visualized mesh at each frame.
4. **Update visualization:** *VR Platform* loads the full meshes from the *Shared data server*, and display only the closest mesh from the user's hand in the virtual environment during modifications.
5. **Validation:** Once the modification is finished, *VR Platform* transmits a chosen parameter value to *VR-CAD Server*. *VR-CAD Server* exports an updated linkage file onto *Shared data server* and *VR Platform*.

## 5. Experiment

In order to assess the efficiency of *ShapeGuide* on a CAD deformation task, we conducted a controlled experiment to compare it with a scroll technique, named *Scroll*. We also wanted to assess the effect of force feedback that could enable participants to “touch” the different parameter values during the modification on both interaction techniques.

The *Scroll* technique allowed participants to manipulate a parameter value of the CAD object by scrolling their arm onto a horizontal axis. The parameter value increased with the motion of the arm toward the right, and vice versa. The direction of *Scroll* was static, which means that it did not rely on the direction of the shape deformation; therefore, the direction of *Scroll* may not be consistent with the shape in most cases.

In this experiment, we limited the number of computation shapes to *N*_*shapes*_ = 10, and parameter step size as Δ*p* = 10 mm to avoid participant related variability regarding these settings and to focus on a fair comparison of the two interaction techniques. As an example, generating the 10 full meshes of *Rear-view mirror* takes between 1.5 and 2.0 s, which was acceptable loading time for participants according to our pilot test.

Consequently, we chose the real space range of the scroll to allow users to reach any of the ten available alternative parameters values from a one hand motion (0.5 m). The step in that range (real size of 5 cm), equally distributed on the horizontal axis, allowed to easily perform parameter value modification with stable force feedback from the gathered results of a pilot test. Also, we provided visual feedback displaying the corresponding modified shape during the manipulation. For stability purposes, the user's hand was snapped onto the scroll axis using attractive force feedback.

From our assumptions based on the related works and some first pilot tests, we formulate the following hypotheses:

**H1:** participants achieve the deformation task faster with *ShapeGuide* than with *Scroll,*
**H2:** participants are more likely to start the deformation in the correct direction with *ShapeGuide* than with *Scroll* because of the consistency of the gesture with the deformation direction,
**H3:** participants prefer *ShapeGuide* to *Scroll,*
**H4:** the magnetic force feedback helps participants to reach the desired parameter values with more precision, especially with *ShapeGuide*.

### 5.1. CAD part

**Figure 6:**
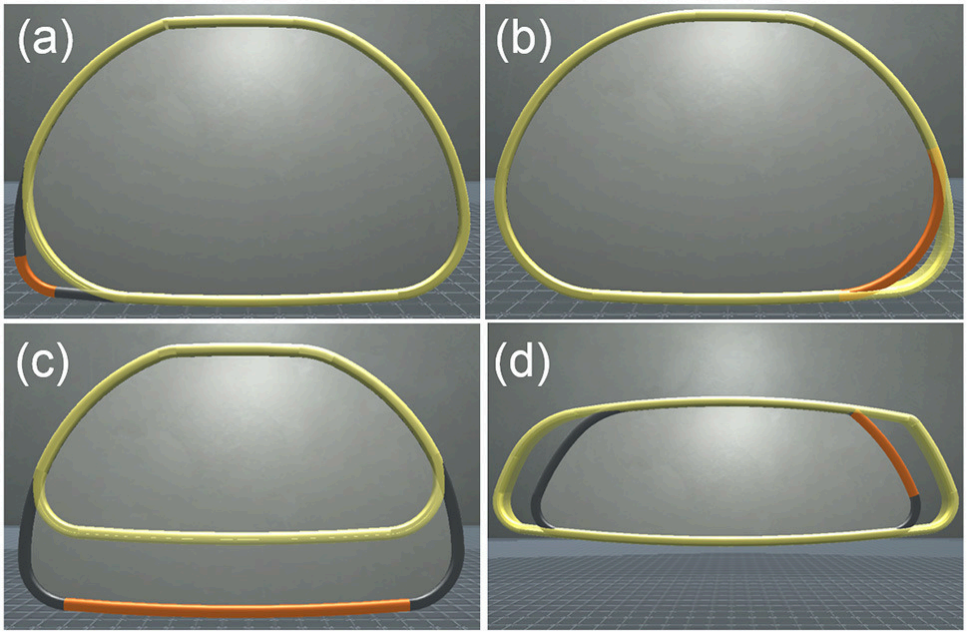
Four examples of the experimental task: orange parts had to be modified to reach the yellow targets. **(a)** *LeftBottomCorner*, +40 mm difference from initial radius. **(b)** *RightBottomCorner*, –30 mm difference from initial radius. **(c)** *Bottom*, –40 mm difference from initial length. **(d)** *RightSide*, +30 mm difference from initial length.

We used four parts of the *Rear-view mirror* (Figure 1) for the deformation task: *LeftBottomCorner, RightBottomCorner, Bottom* and *RightSide* (Figure 6). In *Scroll* condition, shape evolution was consistent with user's hand motion in *RightSide* and *LeftBottomCorner*: right-hand motion led to shape deformation toward the right for both parts. On the contrary, it was inconsistent in *RightBottomCorner* and *Bottom*: right-hand motion led to shape deformation toward the left for the *RightBottomCorner* and toward the bottom for the *Bottom*. We chose these four parts (two consistent ones and two inconsistent ones) to analyze how consistency between the interaction and the deformed shape effects on the modification task.

Moreover, we applied scale factor of 3 on the virtual scene to suit to our experimental VR setup. After scaling, the real-world perceived width of the *Rear-view mirror* was 75 cm, and the distance between two sub-part meshes computed by *ShapeGuide* was from 0.5 cm for the corners up to 6 cm for the side parts. Consequently, we chose two corners to investigate precision issues, and two side parts to assess the efficiency of both techniques since the distance between consecutive shapes with *ShapeGuide* was similar to the *Scroll* step size.

### 5.2. Method

The experiment had a [2 × 2 × 4] within-subject design with the three following factors:

- Technique, with two levels: *ShapeGuide* and *Scroll*.
- Feedback, with two levels: *NoForce* for which the magnetic force feedback is not available, and *Force* for which the magnetic force feedback is available.
- Part, with four levels: the four deformable parts of *Rear-view mirror* (*LeftBottomCorner, RightBottomCorner, Bottom* and *RightSide*).

The techniques used in each Technique×Feedback condition can be described as follow:

- *Scroll, NoForce*: the CAD part is modified with a horizontal scroll of users' hand.
- *Scroll, Force*: in addition to *Scroll*, the magnetic force feedback attracts the users' hand to some attractive points distributed on the horizontal axis. Users can thus feel each discrete parameter value.
- *ShapeGuide, NoForce*: the CAD part is modified in the direction of users' gesture according to the *ShapeGuide* algorithm.
- *ShapeGuide, Force*: in addition to *ShapeGuide*, the magnetic force feedback attracts the users' hand to the closest shape proposed by the *ShapeGuide* algorithm. Users can thus feel each possible shape corresponding to a specific parameter value.

Technique and Feedback are the two main factors, and trials are grouped by Technique×Feedback. The order of Technique×Feedback was counterbalanced across participants using a balanced Latin Square; the order of Part was counterbalanced for each Technique×Feedback.

### 5.3. Hardware and software

The experiment was carried out within a CAVE system, composed of four back-projected stereoscopic screens: 4.8 × 2.7m (front & floor) and 2.7 × 2.7m (left and right). The resolution of each screen is 1,920 × 1,080 pixels. This CAVE is controlled by a cluster of 5 PCs (4 PCs for graphic rendering and one as a master of synchronization) running Windows. *ART* infrared tracking system^2^ was used to track the orientation and position of the participants' head to compute the adaptive view of the virtual environment.

**Figure 7:**
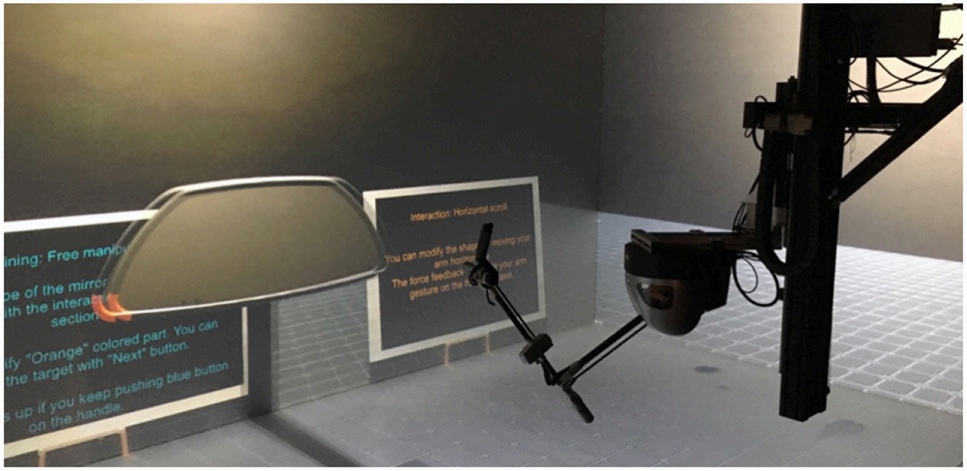
Experimental set-up: CAVE system and *Scale1*.

During the experiment, virtual manipulations and selections were performed using a 9 DoF force feedback capable device, named *Scale1* from *Haption*^3^, which compounds a *Virtuose* (6 DoF haptic device) on a 3 DoF carrier (Figure 7). When the participants move inside the CAVE system while grabbing the handle of the *Virtuose*, the carrier automatically follows them and moves to the most convenient position to let them interact freely anywhere in the CAVE system. *VH Server* runs on Windows PC, controlling the *Scale1*. All PCs are connected through the same local network.

For graphics rendering, *Unity*^4^ simulates the virtual environment, and *MiddleVR for Unity*^5^ manages clustering rendering and computes adaptive view with tracking data.

### 5.4. Participants

We recruited 16 participants, aged between 20 and 63 (11 men and 5 women). Only one person was left-handed. 11 participants had experience of VR system (mostly head-mounted display or CAVE system), and 9 out of 11 participants had used a haptic device before. 2 participants use 3D modeling software on a daily basis (*Blender*^®^, *SketchUp*^®^ and *CATIA V5*^®^), 2 on a weekly basis (*3DS Max*^®^), 2 on a yearly basis (*SolidWorks*^®^, *Maya*^®^ and *123D Design*^®^) and 10 almost never.

### 5.5. Ethics approval

An ethics approval was not required at the time the research was conducted as per our Institution's guidelines and national regulations. However, participants were recruited and treated in accordance with the principles outlined in the Declaration of Helsinki. All participants signed consent forms that details: purpose and procedures, risks and benefits, voluntary participation (they were free to discontinue their participation at any time, without providing a reason and without penalty) and data confidentiality. All collected data were anonymized.

### 5.6. Deformation task

**Figure 8:**
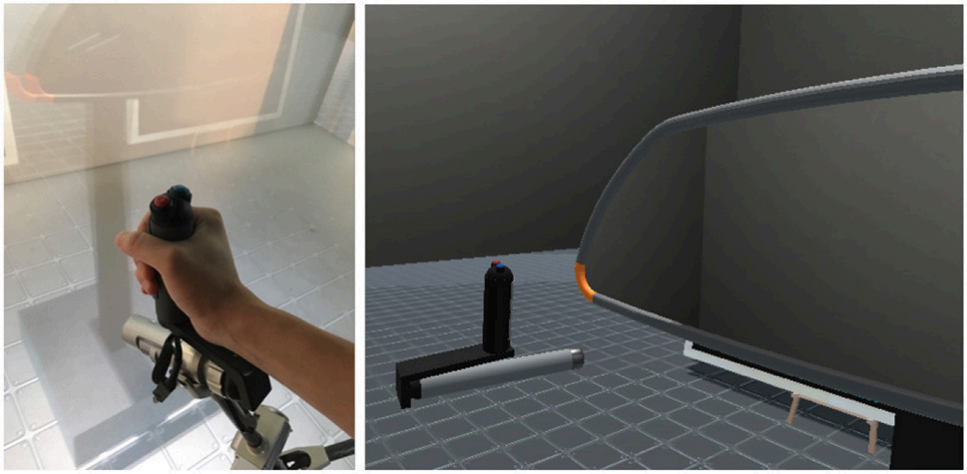
Haptic arm handle of *Scale1* **(left)** and its visual proxy **(right)**. 3D representation of the virtual handle is co-located with the actual one in CAVE system.

We asked participants to perform a deformation task of the *Rear-view mirror*. A virtual representation of a *Virtuose* handle was displayed in the virtual environment (Figure 8). This virtual handle was co-localized with an actual handle of *Scale1* in the CAVE system. This virtual handle was used as an interaction pointer allowing the users to interact with the *Rear-view mirror*.

The deformation scenario was composed of the following steps:

- *Selection* of a part: the participants could select the part by pressing a button on the handle while the virtual handle was colliding on the surface.
- *Modification*: after selection, the scenario automatically switched to modification mode. The participants could start switching between possible shapes by their hand motion after some waiting time for the shape computation. Once they reached the desired 3D shape, they could validate the deformation by pressing the same button once again.

For each trial, participants had to deform the *Rear-view mirror* to fit a target shape, i.e., from an initial parameter value to a targeted parameter value. Only this part of *Rear-view mirror* was modifiable at each trial, colored with Orange (Figure 8). The targeted shape was displayed with a transparent yellow color (Figure 6). If participants failed to deform the shape with the correct targeted parameter value, they had to select the same part and attempt to deform the shape again. We counted this as an error. Once participants achieved the task, the next targeted shape appears. The participants were instructed to accomplish the task as fast as possible.

### 5.7. Procedure

Participants were welcomed and given paper instructions on how to perform the deformation task. They walked then inside of the CAVE system, followed by an instructor who explained them how to operate *Scale1*.

For each Technique×Feedback condition, the participants first performed a training phase before the experimental test. The training phase was composed of two steps:

- *Interaction training*: the participants could deform a part of the *Rear-view mirror* as many time as they wanted to learn the interaction technique.
- *Task training*: the participants accomplished deformation tasks for each one of the 4 Parts of the *Rear-view mirror* to learn the deformation task.

In the experimental test, the participants performed 16 trials: 4 repetitions for each one of the 4 Parts of the *Rear-view mirror*. For each repetition, the initial and targeted parameter values were different. We controlled the offset between the initial and targeted parameter values to have the deformation in both directions and to have comparable parameter modifications for each Part. The order of the different offsets was chosen in a random order among –40, –30, +30, and +40. The same initial and targeted parameter values were used for each Technique × Feedback condition and presented to participants in a random order to avoid learning effect between conditions.

The participants were encouraged to take a break after each block of trials corresponding to a Technique×Feedback condition. At the end of the experiment, the participants filled out a questionnaire. The whole experiment lasted around 40 min including the time to fill out the questionnaire.

### 5.8. Data collection

We registered 1024 trials: 2 Techniques × 2 Feedbacks × 4 Parts × 4 repetitions × 16 participants. For each trial, we logged the time, the evolution of the parameter value and the number of attempts to complete the task. From this data, we extracted four different measures:

- *Task Completion Time (TCT):* the *TCT* measured the total duration of the modification step during the deformation task. The measure started when the participants selected the part to deform and when all possible shapes were loaded. It stopped when participants validated the deformation with the correct parameter value. If the participants made some errors, the *TCT* aggregated the time of the different attempts to reach the correct parameter value. The selection step was not considered since it was similar to all the conditions.
- *Wrong Direction Starts (WDS):* we considered that the participants started their motion in the wrong direction if they started by deforming the part in the opposite direction to the targeted parameter/shape. The *WDS* rate was computed according to the total number of attempts required to achieve the task. For example, if a participant made one error and if the motion of the first attempt were in the correct direction, while the motion of the second was in the wrong direction, the *WDS* rate would be of 0.5 for this trial.
- *Overshoots:* an overshoot was counted when the participants reached the targeted parameter/shape, but continued their gesture further away to a higher or smaller parameter value. Several overshoots can be accumulated during one attempt. Consequently, the number of *Overshoots* is the mean value of overshoots over all the attempts of the same trial. For example, if a participant made one error and if she did 2 overshoots on the first attempt and 3 on the second, the number of *Overshoots* would be of 2.5 for this trial.
- *Errors:* the number of *Errors* was computed from the number of wrong parameter/shape selections in a trial (i.e., number of attempts minus 1).

Finally, the questionnaire assessed the participant preferences. It was designed based on the NASA Task Load Index (TLX) (Hart and Staveland, 1988). *Effort* was replaced by the *Difficulty* to achieve the task, and Time pressure was not asked. We included one extra factor: *Consistency* (i.e., *Did you find the interaction technique consistent with the shape deformation?*). Consequently, the factors of the questionnaire were *Mental Demand, Physical Demand, Difficulty, Frustration Experienced, Consistency*, and *Performance Level*. Participants had to grade each factor using a 5-point Likert scales, and they could also give open-ended comments.

## 6. Results

### 6.1. Task completion time

To minimize noise in our data, we averaged the *TCT* of the 4 repetitions for each Technique × Feedback × Part condition. We tested *TCT* for normality on the whole aggregated data set using a Shapiro-Wilk W test^6^ and found that it was not normally distributed^7^. We tested for goodness-of-fit with a log-normal distribution using Kolmogorov's *D*-test, which showed a non-significant result. Therefore, we ran the analyses using the log-transform of *TCT*, as recommended by Robertson and Kaptein (2016), p. 316. In addition, we did not find any significant learning effects due to technique presentation order.

**Figure 9:**
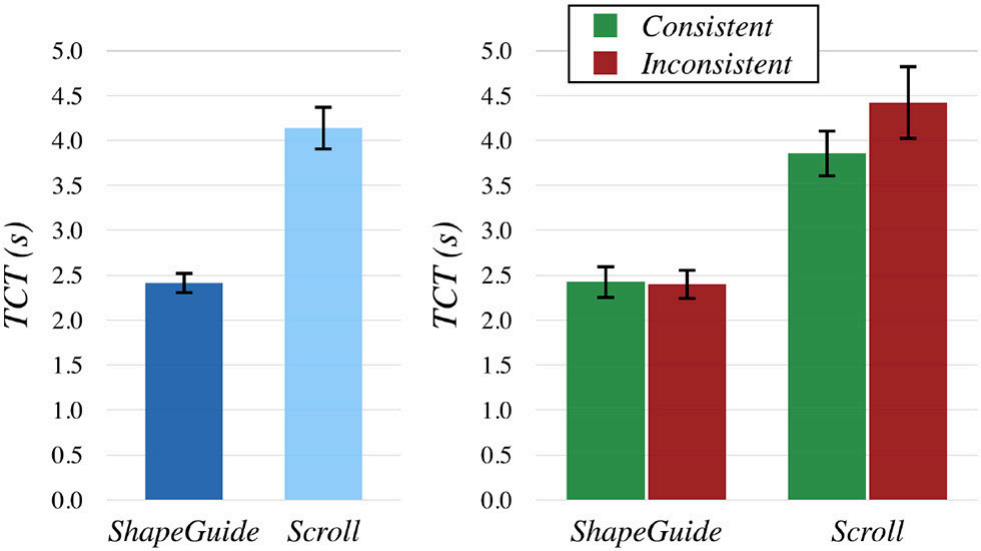
Mean *TCT* by Technique **(left)** and Technique×Consistency **(right)**. Error bars show 95% the confidence intervals (CI).

We ran an analysis of variance on *TCT* with the model Technique×Feedback×Part×Rand(PARTICIPANT) with a REsidual Maximum Likelihood (REML) analysis. The result of the full factorial analysis revealed significant effects of Technique [*F*(1, 225) = 394.20, *p* < 0.0001] and Part [*F*(3, 225) = 8.90, *p* < 0.0001], but no significant effect of Feedback, as well as no significant interaction effects between factors. For Technique, a Students *t*-test showed that *ShapeGuide* (avg. 2.41s) was significantly faster than *Scroll* (avg. 4.14s, *p* < 0.0001) (Figure 9, left^8^). For Part, a *post-hoc* Tukey HSD test indicated that the task was significantly longer to achieve for the *RightBottomCorner* (avg. 3.64s) than for the *RightSide* (avg. 2.94s, *p* < 0.0001) and the *Bottom* (avg. 3.18s, *p* = 0.0017). It was also significantly longer for the *LeftBottomCorner* (avg. 3.34s) in comparison to the *RightSide* (avg. 2.94s, *p* = 0.0112).

To check if the consistency between the gesture and the shape deformation had an impact on *TCT*, we also analyzed the data by grouping parts which produced a consistent and an inconsistent deformation motion according to the gesture direction with the *Scroll* technique. *Rightside* and *LeftBottomCorner* were considered as consistent, *RightBottomCorner* and *Bottom* as inconsistent. We thus ran a REML analysis on *TCT* with the model Technique × Feedback × Consistency × Rand(PARTICIPANT) with Consistency as a factor with two levels: *Consistent* and *Inconsistent*. We observed the same main effects than in the previous analysis, but we detected an additional interaction effect of Technique × Consistency [*F*(1, 233) = 4.36, *p* = 0.038] (Figure 9, right). A *post-hoc* Tukey HSD test indicated that the task was significantly longer to achieve with *Scroll* on *Inconsistent* parts (avg. 4.42s) than with *Scroll* on *Consistent* parts (avg. 3.86s, *p* = 0.0328). Both *Scroll* conditions were also significantly longer than both *ShapeGuide* conditions (*p*'s < 0, 0001).

### 6.2. Wrong direction starts

**Figure 10:**
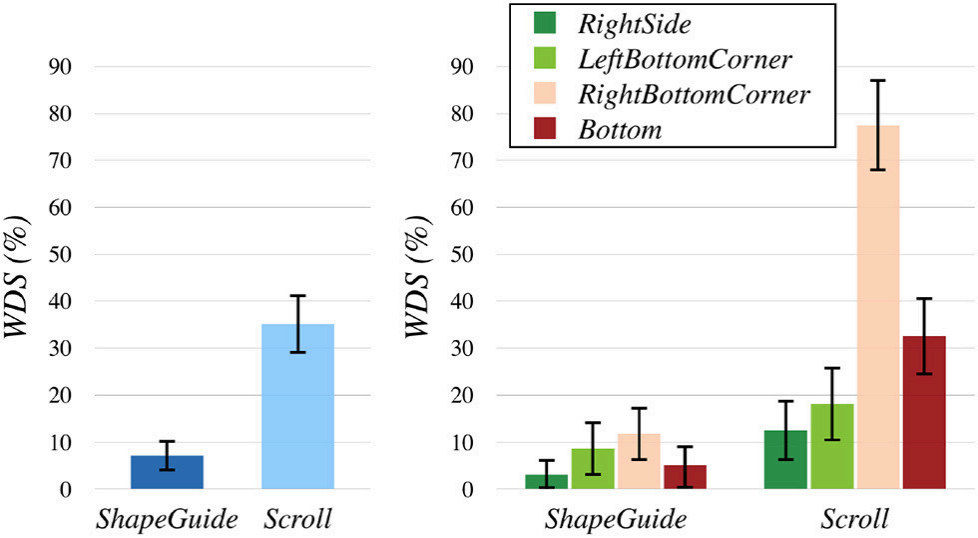
*WDS* rates by Technique **(left)** and Technique × Part **(right)**.

In conformity with the nature of such count data, the *WDS* rate did not follow either normal or log-normal distribution. Consequently, we computed the mean *WDS* rates of each participant by levels for each factor and we used non-parametric tests to compare these values. For Technique, a Wilcoxon Signed Rank test showed that *ShapeGuide* led to significantly fewer *WDS* (7% of *WDS*) than *Scroll* (35% of *WDS*, *p* < 0.0001) (Figure 10, left). For Feedback, a Wilcoxon Signed Rank test did not reveal any significant differences. For Part, a Friedman test was conducted and rendered a Chi-square value of 31.38 which was significant (*p* < 0.0001). A *post-hoc* analysis detected that the *RightBottomCorner* induced significantly more *WDS* (45% of *WDS*) than the *RightSide* (8% of *WDS*, *p* < 0.0001), the *LeftBottomCorner* (13% of *WDS*, *p* < 0.0001) and the *Bottom* (19% of *WDS*, *p* = 0.0266).

In addition, we used the Aligned Rank Transform (ART) procedure proposed by Wobbrock et al. (2011) to analyze the data and have hints of the interaction effects between factors. Data were aligned with the ARTool, and we ran a REML analysis on *WDS* rate with the model Technique×Feedback×Part×Rand(PARTICIPANT). It confirmed the main effects described previously and detected an interaction effect of Technique × Part [*F*(3, 225) = 40.23, *p* < 0.0001] (Figure 10, right). A *post-hoc* analysis revealed that *Scroll* on the *RightBottomCorner* led to significantly more *WDS* than all the other combinations (*p*'s < 0, 0001). It showed that *Scroll* on the *Bottom* induced significantly less *WDS* than *Scroll*+*RightBottomCorner*, but significantly more *WDS* than all the other combinations (*p*'s < 0, 04).

### 6.3. Overshoots

**Figure 11:**
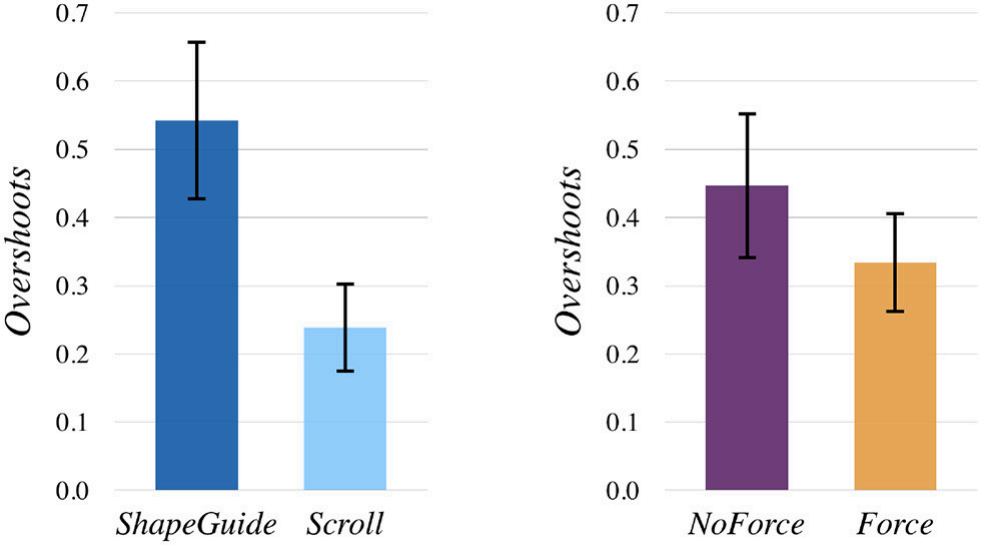
Mean *Overshoots* by Technique **(left)** and Feedback **(right)**.

A similar analysis to the one for *WDS* rate was conducted for *Overshoots*. For Technique, a Wilcoxon Signed Rank test showed that *ShapeGuide* led to significantly more *Overshoots* (avg. 0.54) than *Scroll* (avg. 0.23, *p* < 0.0001) (Figure 11, left). However, for Feedback, a Wilcoxon Signed Rank test revealed that *Force* significantly reduced the number of *Overshoots* (avg. 0.33) in comparison to *NoForce* (avg. 0.45, *p* = 0.0240) (Figure 11, right). For Part, a Friedman test was conducted and rendered a Chi-square value of 14.71 which was significant (*p* = 0.0021). A *post-hoc* analysis detected that the *RightSide* induced significantly less *Overshoots* (avg. 0.26) than the *RightBottomCorner* (avg. 0.52, *p* = 0.0023) and the *LeftBottomCorner* (avg. 0.45, *p* = 0.0189).

**Figure 12:**
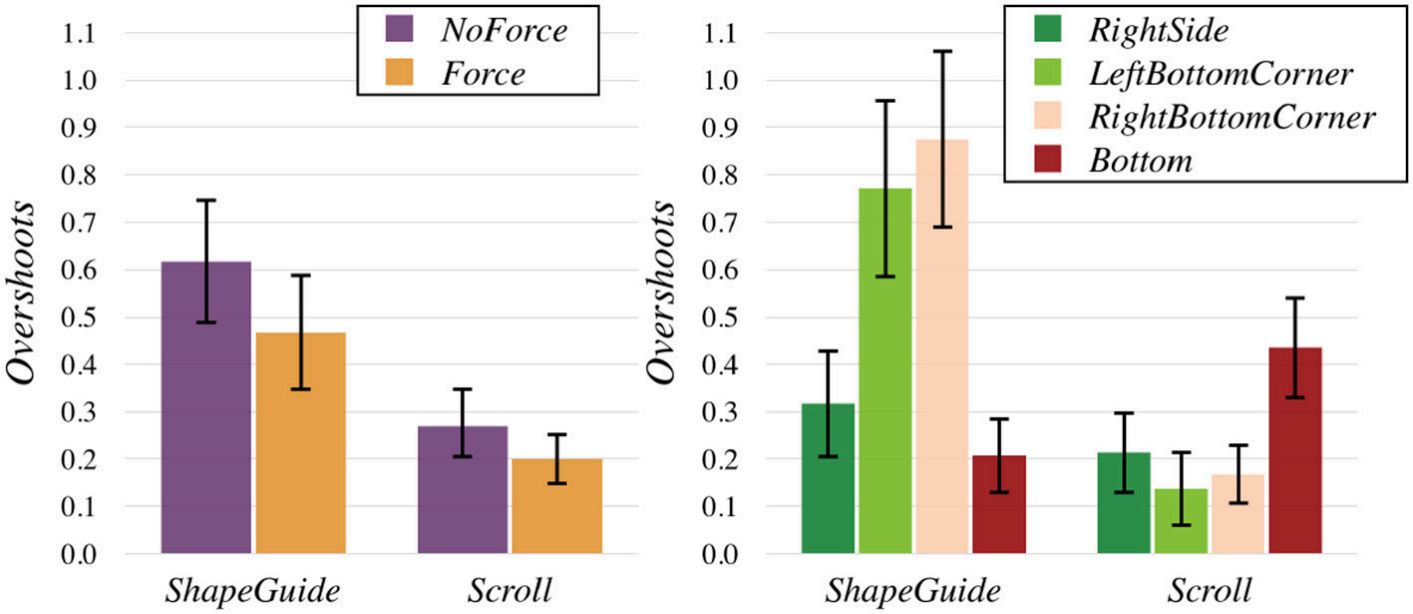
Mean *Overshoots* by Technique × textscFeedback **(left)** and Technique×Part **(right)**.

We also ran a REML analysis on *Overshoots* with the model Technique×Feedback×Part×Rand(PARTICIPANT) after that the data was aligned with the ART procedure. It confirmed the main effects described previously and detected an interaction effect of Technique×Feedback (*F*(1, 225) = 4.01, *p* = 0.047) (Figure 12, left) and an interaction effect of Technique×Part [*F*(3, 225) = 36.60, *p* < 0.0001] (Figure 12, right). For Technique×Feedback, *ShapeGuide* with *Force* seemed to significantly reduce the number of *Overshoots* (avg. 0.47) in comparison to *ShapeGuide* with *NoForce* (avg. 0.62, *p* = 0.0103). *ShapeGuide* with and without force was also significantly different from both *Scroll* combinations (*p*'s < 0, 002). For Technique×Part, *ShapeGuide* on both the *RightBottomCorner* and the *LeftBottomCorner* led to significantly more *Overshoots* than all the other combinations (*p*'s < 0.0015). *Scroll*+*Bottom* was also significantly difference from *Scroll*+*RightBottomCorner* and *Scroll*+*LeftBottomCorner* (*p*'s < 0.03).

### 6.4. Errors

We used the same method than for *WDS* and *overshoots* to analyze the number of *Errors*, but we did not observe any significant differences for Technique, Feedback and Part.

### 6.5. Subjective questionnaire

**Figure 13:**
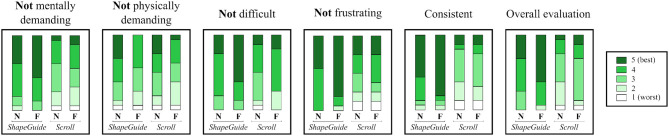
Participant ratings by Technique×Feedback for not *mentally demanding*, not *physically demanding*, not *difficult* to use, not *frustrating, consistent* and *overall evaluation* on a 5-point Likert scale (5 is best, 1 is worst). **N**, *NoForce* and **F**, *Force*.

In the questionnaire, participants had to grade each one of the 4 Technique×Feedback combinations on a 5-point Likert scale. To avoid confusion for participants, we phrased the questions so that they always had to give a good grade if they appreciated the interaction technique for all the criteria. Consequently, we asked them if they found the interaction technique not *mentally demanding*, not *physically demanding*, not *difficult* to use, not *frustrating* and *consistent* with the deformation of the shape (from 1: strongly disagree to 5: strongly agree). They also had to give an *overall evaluation* (from 1: bad to 5: good). Figure 13 illustrates the results of the subjective questionnaire.

To analyze the data, we computed the mean grades of each participant by levels for Technique and Feedback. We used a non-parametric Wilcoxon Signed Rank test to compare each level. For Technique, *ShapeGuide* was perceived less *mentally demanding* (avg. 4.28 vs. 3.16, *p* = 0.0046), less *frustrating* (avg. 4.56 vs. 3.44, *p* = 0.0098), less *difficult* to use (avg. 4.28 vs. 3.56, *p* = 0.0093) and more consistent (avg. 4.5 vs. 2.88, *p* = 0.0005) than *Scroll*. In general, *ShapeGuide* was also preferred by the participants in comparison to *Scroll* (avg. 4.28 vs. 3.06, *p* = 0.0040). For Feedback, *Force* was considered more physically demanding in comparison to *NoForce* (avg. 3.06 vs. 3.62, *p* = 0.0293).

## 7. Discussion

The results of the experiment provide evidence that *ShapeGuide* technique significantly increases user performance on parametric modification of CAD data in comparison to a one-dimensional scroll technique used as a baseline. More precisely, participants were able to achieve the deformation task 42% faster with *ShapeGuide* than with the *Scroll* technique, which supports **H1**.

This improvement can be explained by a better consistency between shape deformation and user hand motion with *ShapeGuide*. In particular, we observed that *ShapeGuide* reduced of 80% the chance that participants move their hands toward the wrong direction at the beginning of their gesture, in comparison to *Scroll*. This is especially true on parts which produce an inconsistent deformation motion according to the gesture direction (*RightBottomCorner* and *Bottom*): *Scroll* on the inconsistent parts led to significantly more *wrong direction starts* than on the other parts. This has a certain impact on performance since the task with *Scroll* took longer on *Inconsistent* parts than on *Consistent* parts. Conversely, we did not observe any similar effects with *ShapeGuide*: the *WDS* rate was more consistent between parts (Figure 10, right), and there was no time difference between *Inconsistent* and *Consistent* parts. This is beneficial for users because they can thus expect the same behavior in every part. The subjective questionnaire also confirmed that the participants perceived *ShapeGuide* as more consistent that the *Scroll* technique. For all these reasons, **H2** is validated.

In the subjective questionnaire, most of the participants reported that they preferred *ShapeGuide* to the *Scroll*, which supports **H3**. In particular, they found *ShapeGuide* less mentally demanding, less frustrating and less difficult to use. This can be explained by the better consistency of *ShapeGuide*: users have to think less about the direction toward which they need to move their hand, and they can more easily predict what will be the result of their actions. The efficiency of *ShapeGuide* has probably also a positive impact on the user preference.

The main limitation of *ShapeGuide* is that it significantly increases the number of overshoots in comparison to the *Scroll* technique. We observed this phenomenon mainly on the corner of the *Rear-view mirror*: *ShapeGuide* on both the *RightBottomCorner* and *LeftBottomCorner* led to significantly more *Overshoots* than *ShapeGuide* on the two other parts and than *Scroll* on any parts (Figure 12, right). However, *ShapeGuide* on the *RightSide* and *Bottom* was not significantly different from the *Scroll* on any parts. The distance between consecutive shapes proposed by *ShapeGuide* is smaller for the corner than for the other parts. Consequently, a possible explanation for the higher number of *Overshoots* is that it is harder to reach the desired shape with *ShapeGuide* when this distance is small: users are more likely to encounter unwanted switches between the targeted shape and the next one if the gap between these consecutive shapes is small. On the contrary, the distance between two consecutive parameter values on the scroll axis is always the same for the *Scroll* technique. This distance is especially defined to avoid unwanted switches; therefore *Overshoots* have less chance to occur with the *Scroll* technique less than *ShapeGuide* (see section 5.1).

The introduction of scale management into *ShapeGuide* may significantly reduce *Overshoots*. To implement such function, the scale value should be computed to guarantee that the minimal distance between available shapes fits human hand motion precision capabilities.

With respect to this issue about *Overshoots*, the results show that magnetic force feedback can be a effective solution to reduce the number of *Overshoots* and thus, improves the precision of both techniques, and especially *ShapeGuide*. During the experiment, participants achieved the deformation task with 27% fewer *Overshoots* with the magnetic force feedback than without, which supports **H4**. Force feedback also led to a reduction of 24% *Overshoots* for *ShapeGuide*, while the reduction was no significant for the *Scroll* technique. Finally, it seems that the magnetic force feedback has a small drawback: participants perceived both techniques more physically demanding with the magnetic force feedback than without.

In this experiment, we did not let users to choose the number of shapes *N*_*shapes*_, neither the parameter step size Δ*p* nor the target constraint related to his sub-part selection. Indeed, these features are necessary for designers to reshape the model precisely and to address the complex CAD model containing parameters defined by multiple constraints. Our VR-CAD system can easily support these features by providing a user interface for the parameter tuning although the user interaction on that interface have to be carefully designed to consider the usability. Those feature limitations in the experiment eliminated any biases on comparison of the two interaction techniques for the shape modification task as the task completion time should not be affected by these parameter tuning.

**Figure 14:**
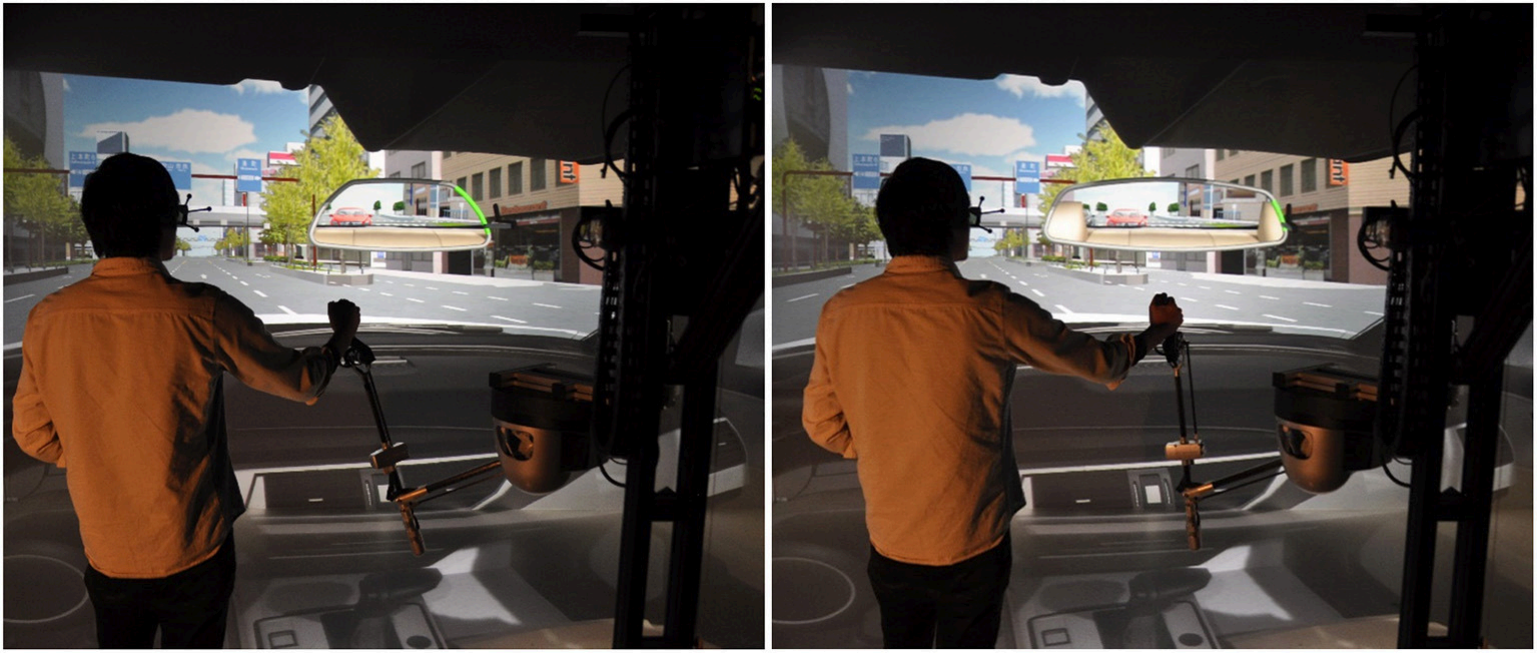
Native CAD data modification in a user-oriented environment. A user modifies the shape of *Rear-view mirror* within a realistic virtual environment of a car's cockpit while perceiving modification impact from a first person perspective in real time.

As a VR scene configuration, we chose to use a simple visual context (Figure 7) to let the users focus on experimental tasks. But, our VR-CAD system supports real-time context switching during the VR session. It would be useful for project members to experience the product considering different contexts specified in PLM, such as end user-oriented environment (Figure 14, Supplementary Material), manufacturing lines, maintenance scenario, external observer. As several experts are involved in immersive product reviews, these environments could facilitate their arguments based on the mutual experience to support their design choices.

In comparison to the work by Coffey et al. (2013), our VR-CAD system can provide users a extensible 3D workspace and support a run-time computation for sampling possible shapes before each modification, instead of pre-processing all the data beforehand.

## 8. Conclusions and future work

This paper presents *ShapeGuide*, a 3D interaction technique allowing users to modify parameter values of native CAD data by directly pushing/pulling its surfaces in an immersive environment. *ShapeGuide* proposes users a set of shape variations by computing several meshes from different parameter values. Consequently, users can select the desired shape from a 3D hand motion with an optional force feedback guidance during shape modification.

We conducted a controlled experiment to investigate the efficiency of *ShapeGuide* for industrial CAD part parameter modifications. We compared it with a one-dimensional scroll technique (*Scroll*), which enables participants to manipulate parameter values with a left-right hand motion. The impact of attractive force feedback to assist the parameter value setting was also investigated for both interaction techniques.

The experiment demonstrated that *ShapeGuide* is faster than the *Scroll* technique for a deformation task. This can be explained by the fact that the shape deformation is more consistent with the users' hand motion when using *ShapeGuide*. As a consequence, most participants preferred *ShapeGuide*.

The experiment also showed that *ShapeGuide* could be less precise in selecting the desired shape when the gap between consecutive shapes is tiny. According to the results, the force feedback is a first solution to enhance the precision of *ShapeGuide* in those cases. Also, we are currently exploring solutions to solve this problem. A work in progress extends the 3D based distance computation approach (see Algorithm 1) by taking into account the orientation of the users' hand regarding a pseudo-normal vector at the closest point on the surface. Furthermore, we plan to scale the scene according to the detected distance between sub-part meshes on shape computation.

Previous research work attempted to decrease the number of iterations between workstations and VR platforms by allowing users to directly modify CAD data during immersive design reviews. From the experiment, we observed that *ShapeGuide* significantly enhanced the previously used scroll-based interaction technique. Through *ShapeGuide*, non-CAD experts can easily access and manipulate parameter values without a deep understanding of the internal organization of CAD data. As a consequence, it reduces misunderstanding or misinterpretation errors that could occur when non-CAD experts specify modification requests to CAD engineers.

In the experiment, we imposed static values of the number of computed shapes and parameter step size for comparison purpose. As we described in section 3.1, users can tune these variables on selection stage. To enable precise parameter value setting while keeping a suitable loading time, we recommend to limit the number of computed shapes according to the system performances combined with a successive selection approach using standard parameter step size (e.g., 1 m, 1 cm, 1 mm).

Some further works need to be done to meet the requirements for the industrial design process. The current implementation of *ShapeGuide* uses a CAVE system and a force feedback device. However, this concept can address heterogeneous VR platforms. For example, we also implemented *ShapeGuide* by using a head-mounted display and its controllers (HTC-VIVE®). Furthermore, we are implementing a collaborative design scenario using *ShapeGuide* to address simultaneous modifications by several experts from remote locations.

## Ethics statement

Experiment did not involve any children, disable people, or any medical related sensible conditions. Our ergonomic study hired 16 participants who declared healthy conditions. All participants filled in ethics related consent forms. Experiment was conducted before the existence of ethic committee of university Paris-Saclay (POLETHIS), but in accordance with the principles outlined in the Declaration of Helsinki. The consent forms are available if required.

## Author contributions

YO and NL worked our almost all of the technical details. YO performed the experiment. CF helped to design the experiment and performed the statistical analysis of the results. NL and PB conceived the original idea. CF and PB supervised the project. All authors discussed the method, experimental design and results, and contributed to the final manuscript.

### Conflict of interest statement

The authors declare that the research was conducted in the absence of any commercial or financial relationships that could be construed as a potential conflict of interest.

## Footnotes

^1^http://vcg.isti.cnr.it/vcglib/

^2^http://www.ar-tracking.com/

^3^https://www.haption.com/en/products-en/scale-one-en.html

^4^http://unity3d.com/

^5^http://www.middlevr.com/middlevr-for-unity/

^6^All analyses except Friedman tests were performed with the *SAS JMP* statistical platform. Friedman tests were performed with R.

^7^We used a significance level of α = 0.05 for all statistical tests.

^8^In all barplots, error bars show the 95% confidence intervals (CI).
